# Supplementary figures and images for: Retrotransposon LINE-1 bodies in the cytoplasm of piRNA-deficient mouse spermatocytes: Ribonucleoproteins overcoming the integrated stress response
Source: PLoS Genet. 2023 Jun 12;19(6):e1010797. doi: 10.1371/journal.pgen.1010797 (PMC10289378; doi:10.1371/journal.pgen.1010797)

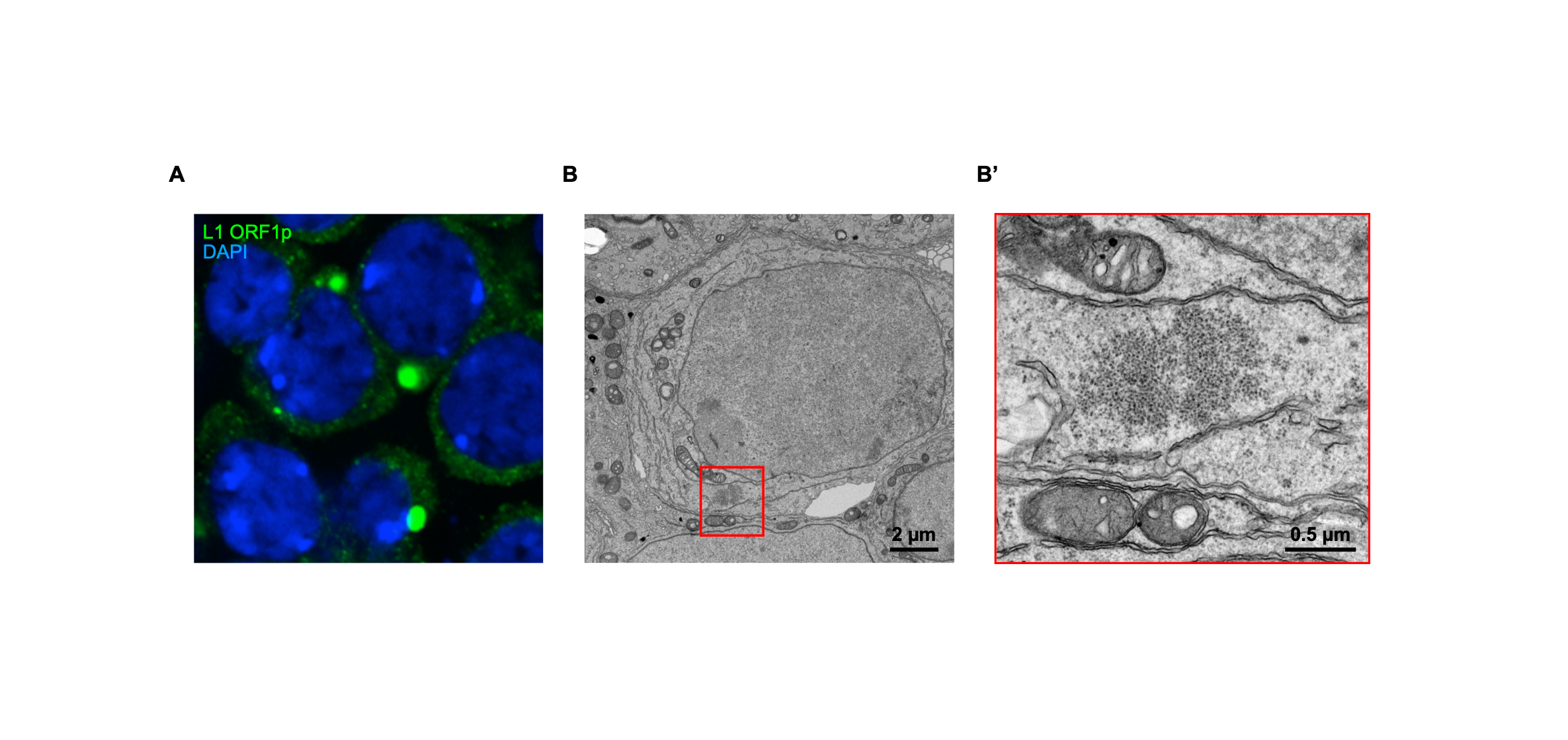

Supplement: S1 Fig — (A) Immunofluorescence staining of L1 ORF1p (green) on BALB/c wild-type spermatocytes showing ORF1p accumulation in small cytoplasmic granules similar to early LBs. (B–B’) Electron micrographs of a BALB/c wild-type spermatocyte harboring a small LB; boxed area in B is magnified in B’. (TIF) [file pgen.1010797.s001.tif]

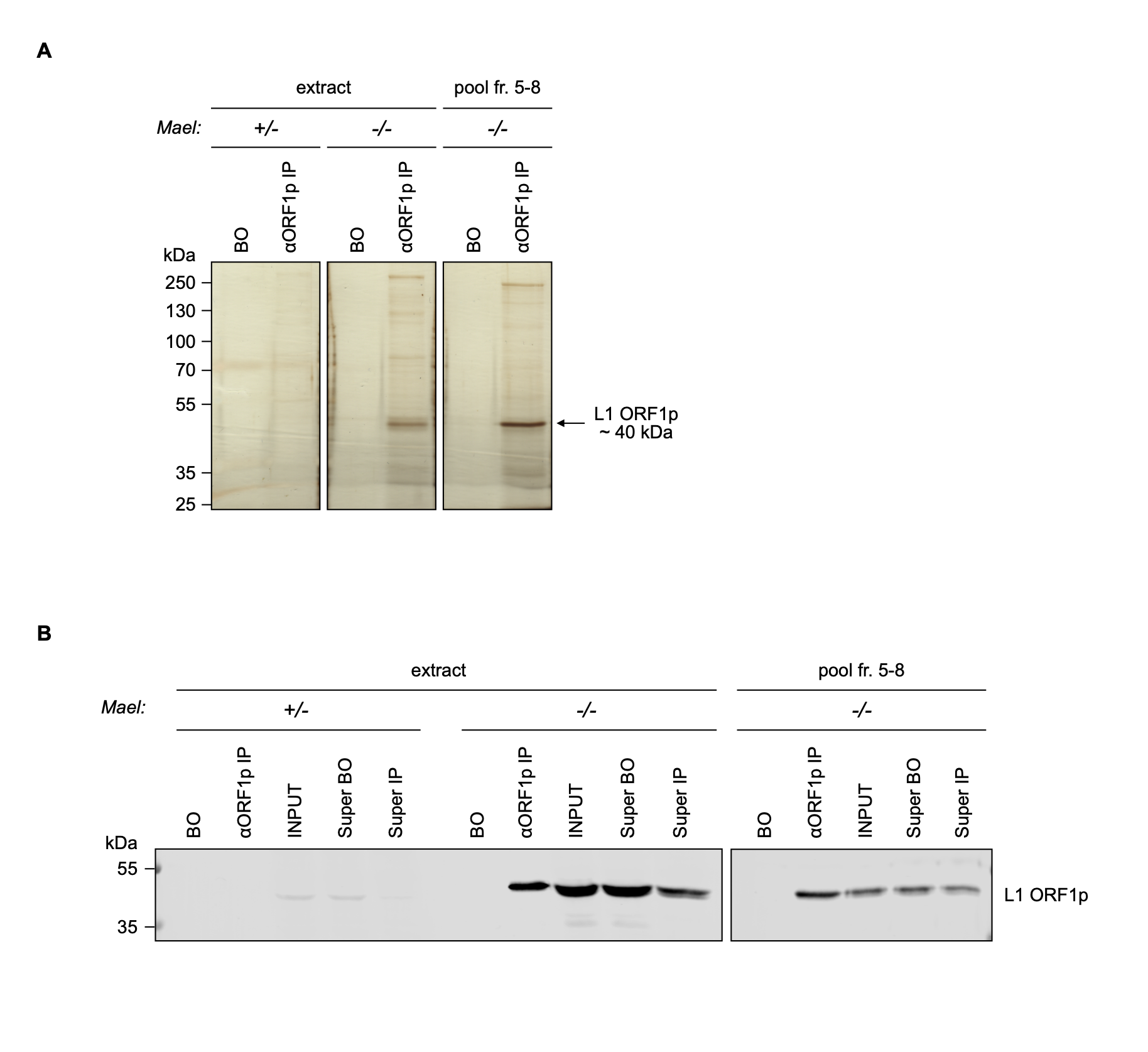

Supplement: S2 Fig — (A) Silver staining and (B) western blot analysis of anti-ORF1p immunoprecipitation samples (IP) from unfractionated and sucrose gradient fractionated (+ EDTA; pool sucrose fractions 5–8) Mael-/- testis extracts. Immunoprecipitations with carrier beads only (BO) were performed in parallel for background estimation; samples obtained from Mael+/- mice are shown as negative controls. Super: supernatant. (TIF) [file pgen.1010797.s002.tif]

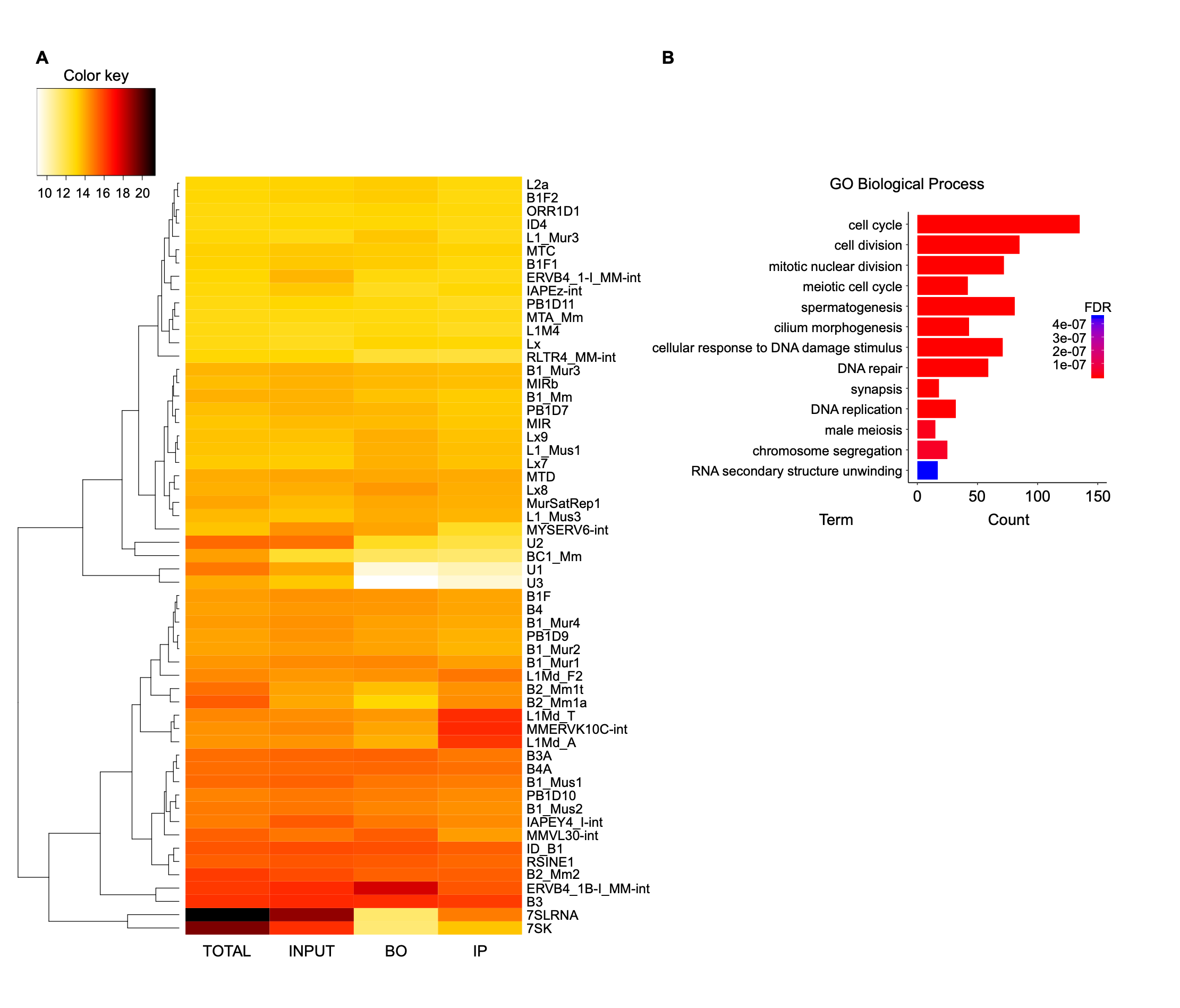

Supplement: S3 Fig — (A) Extensive heatmap of repeat families detected across anti-L1 ORF1p co-immunoprecipitation samples: Mael-/- testis extracts (TOTAL), pooled sucrose fractions 5–8 (INPUT), carrier beads only (BO), ORF1p immunoprecipitated samples (IP). Average expression level of ancestral L1Lx family in TOTAL samples was chosen as the threshold to exclude any repeat with a lower expression from the reported heatmap. For simplicity, low complexity regions, rRNA, tRNA, satellites and simple repeats were also excluded. (B) Gene Ontology analysis (Biological Process) of the set of mRNAs (n = 1347) that were found strongly enriched in IP samples. Barplot shows the 13 most representative enriched GO terms (y axis, Term), with the corresponding number of genes identified per term (x axis, Count). Bars are labeled based on the False Discovery Rate (FDR). (TIF) [file pgen.1010797.s003.tif]

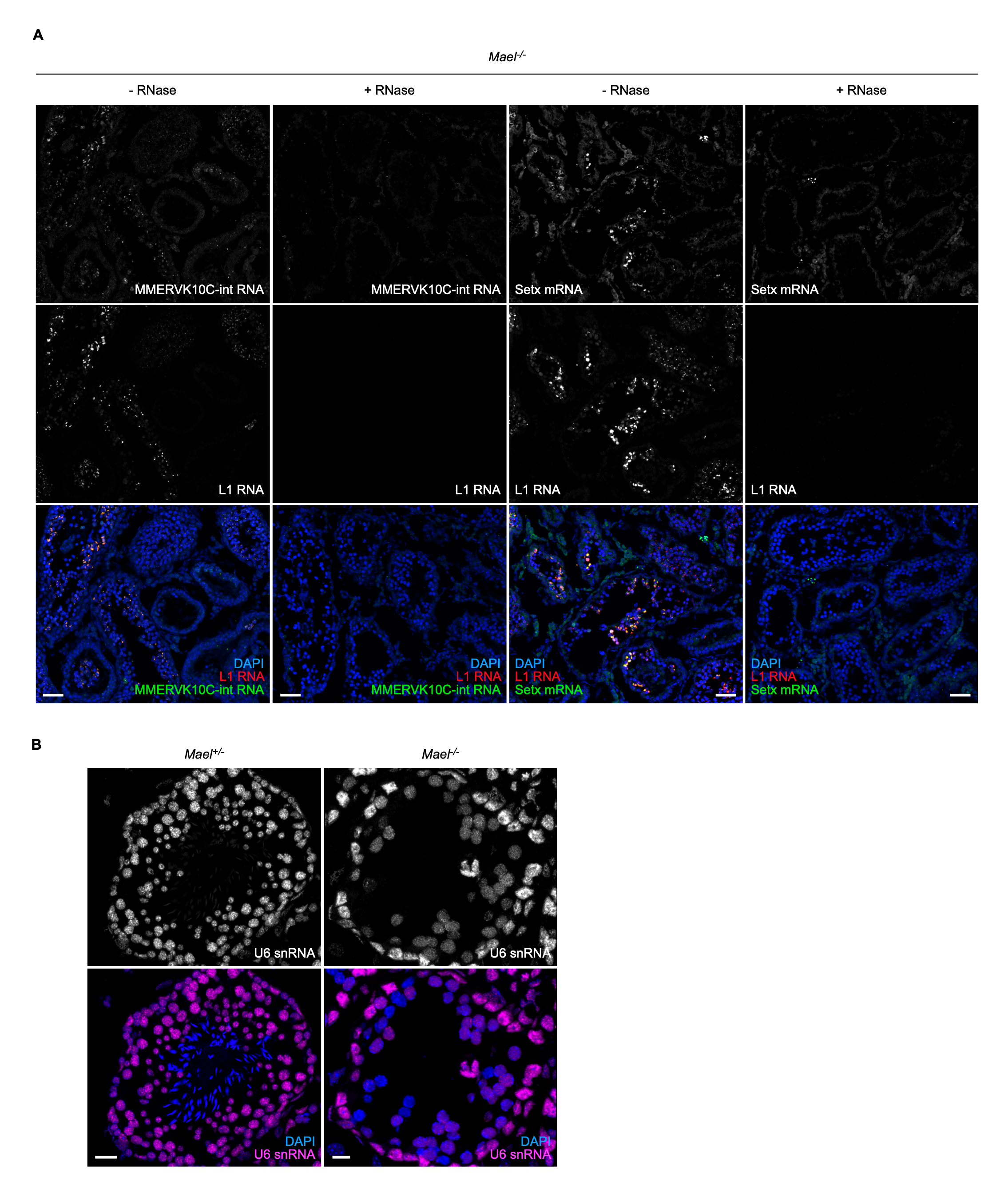

Supplement: S4 Fig — (A) Multiplexed HCR RNA-FISH of MMERVK10c-int and Setx with L1 RNA on Mael-/- testis sections minus (-) and plus (+) RNase A treatment. The RNase treatment is shown as a negative control for effective probe-RNA recognition. Scale bars: 40 μm. (B) HCR RNA-FISH of U6 small nuclear RNA on Mael+/- and Mael-/- testes. U6 small nuclear RNA was chosen as a positive control for its high abundance and strict nuclear localization. As expected, U6 shows strong nuclear signals, independent from the Mael mutation. Scale bars: Mael+/- section: 20 μm; Mael-/- section: 10 μm. (TIF) [file pgen.1010797.s004.tif]

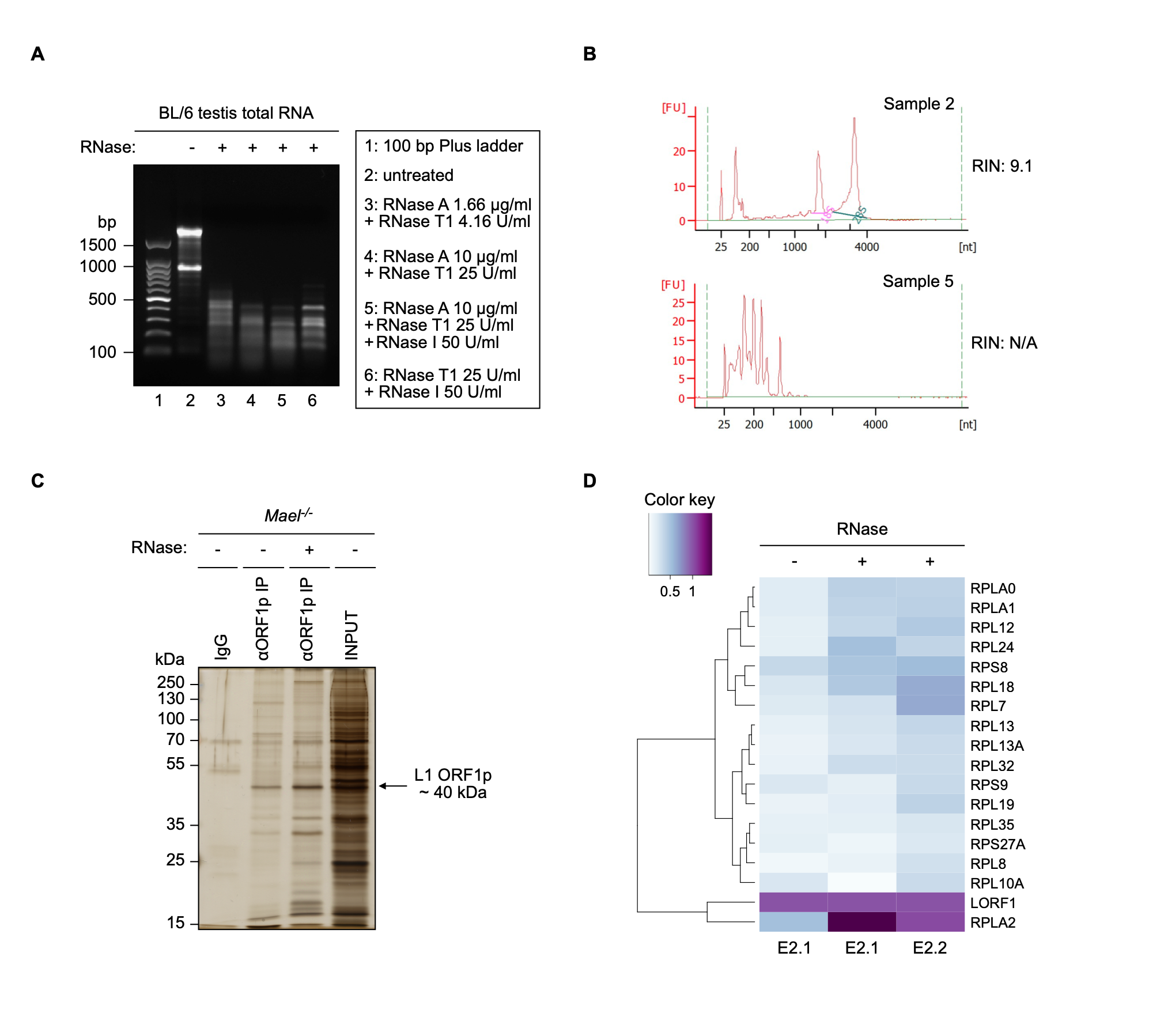

Supplement: S5 Fig — (A) Non-denaturing RNA agarose gel showing multiple RNase treatment conditions (lanes 3–6, see box for details) or untreated BL/6 testis total RNA (lane 2) as a control; the treatment used in lane 5 was chosen for co-immunoprecipitation experiments. Approximately 2 μg of RNA were loaded on each lane. (B) Bioanalyzer traces of RNA samples corresponding to lanes 2 and 5 in panel A. RIN: RNA Integrity Number; scale from 1 (fully degraded) to 10 (intact). (C) Silver staining of anti-ORF1p co-immunoprecipitation samples (IP) from Mael-/- testicular extracts minus (-) and plus (+) the RNase treatment used for lane 5 in panel A. Immunoprecipitation with an isotype IgG served as a negative control; note that the RNase treatment increases immunoprecipitation of ORF1p. (D) Heatmap showing the recovery of ribosomal proteins in anti-ORF1p co-immunoprecipitation samples from Mael-/- testes, minus (-) and plus (+) the RNase treatment used for lane 5 in panel A. Color indicates protein levels; E2.n indicates Experiment 2.replicate n. The relative abundance of each protein in each eluate was obtained by mass spectrometry analysis and further adjusted by dividing the PSM counts by molecular weights (MW); for a sample-to-sample comparison, the obtained PSM / MW ratios were normalized to the ratio obtained for the bait (LORF1). (TIF) [file pgen.1010797.s005.tif]

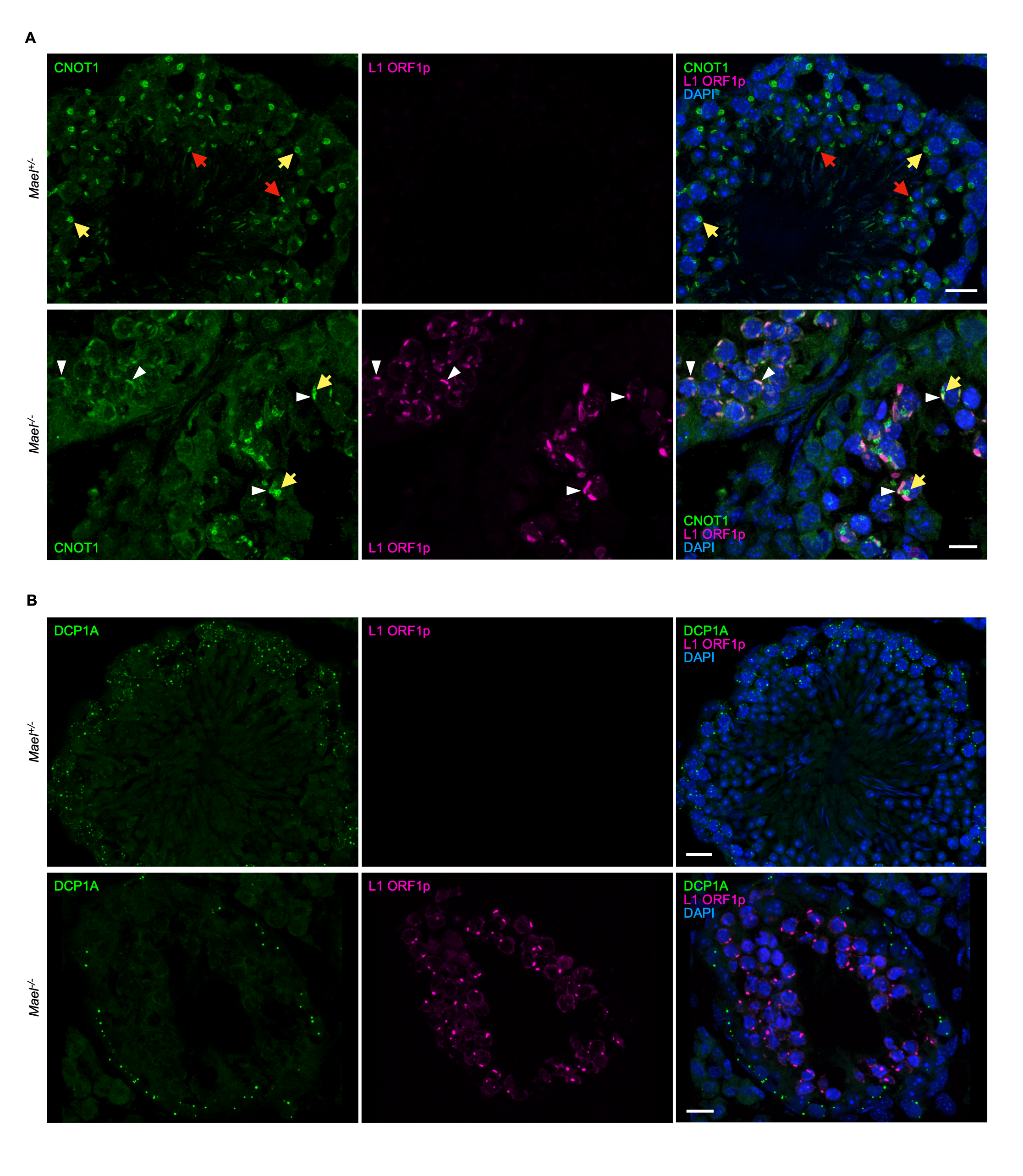

Supplement: S6 Fig — (A) Double immunofluorescence staining of CNOT1 (green) and L1 ORF1p (magenta) in Mael+/- and Mael-/- testes. In Mael+/-, CNOT1 shows aggregation in characteristic round cytoplasmic structures in spermatocytes (top panels, yellow arrows) and in more restricted cytoplasmic areas in round spermatids (top panels, red arrows). Similar structures are also observed in spermatocytes of Mael-/- mice (bottom panels, yellow arrows), where L1 ORF1p is not detected, in addition to some CNOT1 aggregation in LBs (bottom panels, white arrowheads). Scale bars: 15 μm. (B) Double immunofluorescence staining of DCP1A (green) and L1 ORF1p (magenta) in Mael+/- and Mael-/- testis sections. DCP1A shows a cytoplasmic granular pattern that is detected in all spermatogenic cells of Mael+/- control testes (top panels); signal intensities and sizes of granules are variable. In Mael-/- tubules (bottom panels), DCP1A is mostly confined to prominent granules with strong signals in peripheral spermatogonia; weak diffuse DCP1A signal is observed in LBs. Scale bars: 15 μm. (TIF) [file pgen.1010797.s006.tif]

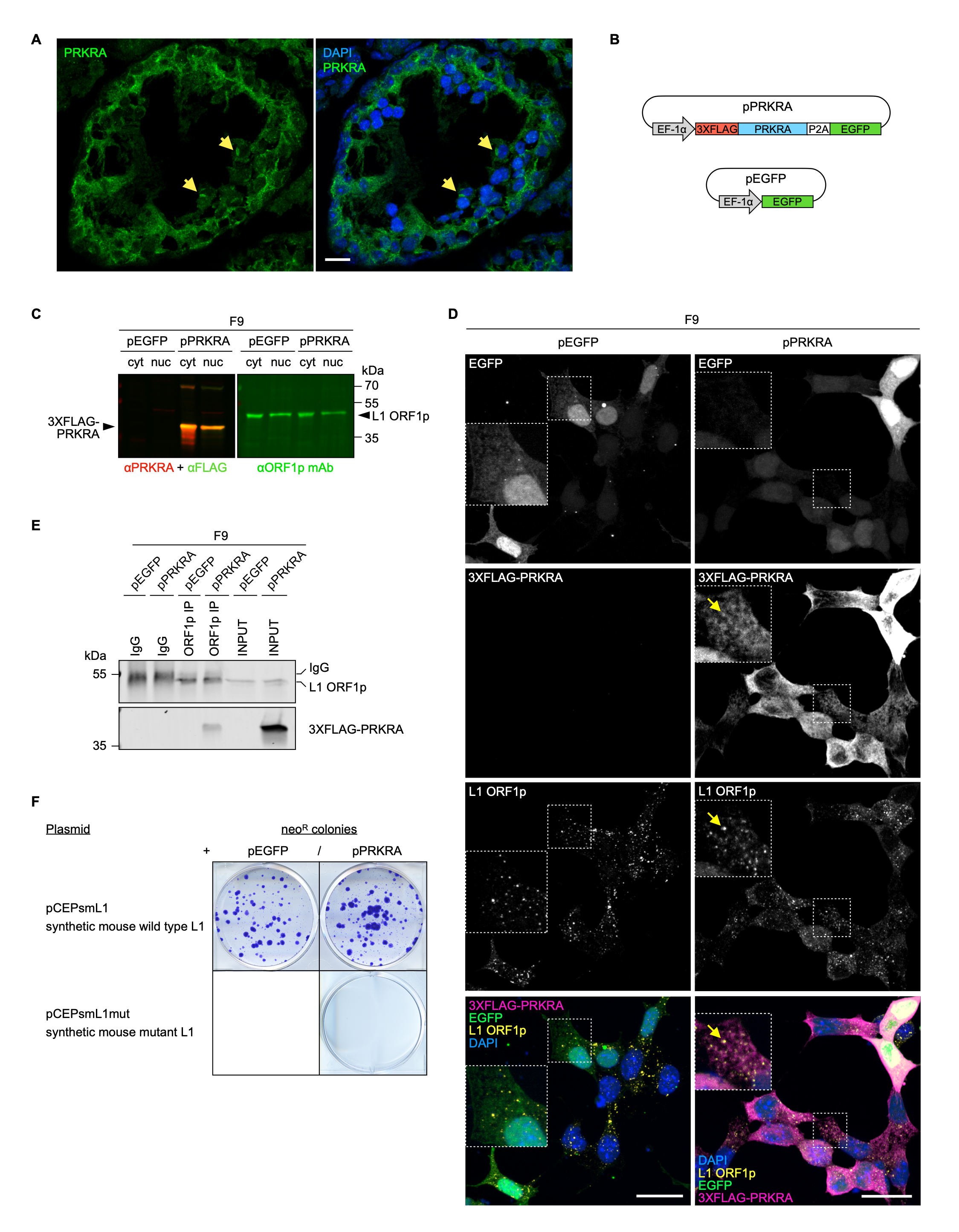

Supplement: S7 Fig — (A) Immunofluorescence staining of PRKRA (green) in Mael-/- testis. PRKRA shows diffuse cytoplasmic signals in both germ (spermatogonia and spermatocytes) and somatic (Sertoli) cells; PRKRA aggregation in LBs-resembling structures in spermatocytes is also detected (yellow arrows). Scale bar: 15 μm. (B) Schematic representation of the pPRKRA plasmid driving the expression of N-terminal 3XFLAG mouse PRKRA followed by the self-cleaving P2A peptide and an EGFP reporter, and its control empty vector pEGFP. (C) Western blot analysis of exogenous PRKRA and endogenous ORF1p in F9 cells transfected with pEGFP or pPRKRA. Exogenous PRKRA was detected by both an anti-PRKRA and an anti-FLAG antibody with overlapping signals (red and green, respectively; left blot) at the expected MW (~ 37 kDa); no detectable perturbation of endogenous ORF1p was observed upon PRKRA overexpression (right blot). cyt: cytoplasmic extract; nuc: nuclear extract. (D) Double immunofluorescence staining of 3XFLAG-PRKRA (anti-FLAG antibody) and L1 ORF1p in F9 cells transfected with pEGFP or pPRKRA. Boxed areas in pEGFP panels are magnified in corresponding insets and identify a cell with a relatively high EGFP expression; besides a major nuclear localization due to an added nuclear localization signal (NLS), EGFP shows a diffuse cytoplasmic distribution independent of L1 ORF1p granules. Boxed areas in pPRKRA panels are magnified in corresponding insets and identify a cell with a moderate expression level of pPRKRA plasmid; exogenous 3XFLAG-PRKRA shows a cytoplasmic distribution that partially overlaps with L1 ORF1p granules (yellow arrow). Scale bars: 20 μm. (E) Western blot analysis of anti-ORF1p co-immunoprecipitation samples obtained from F9 cells transfected with pEGFP or pPRKRA; 3XFLAG-PRKRA co-precipitates with endogenous ORF1p. Immunoprecipitations with an isotype IgG served as negative controls. (F) Additional positive and negative controls for L1 retrotransposition assay. Synthetic mouse wild typ [file pgen.1010797.s007.tif]
