# Supplementary material for: Retrotransposon LINE-1 bodies in the cytoplasm of piRNA-deficient mouse spermatocytes: Ribonucleoproteins overcoming the integrated stress response
Source: PLoS Genet. 2023 Jun 12;19(6):e1010797. doi: 10.1371/journal.pgen.1010797 (PMC10289378; doi:10.1371/journal.pgen.1010797)
Supplement: Attachment — Submitted filename: Response to Reviewers.pdf [file pgen.1010797.s013.pdf]

Authors' Response to Reviewers.

Reviewer #1: In this manuscript by De Luca et. al. the authors investigate the composition of LINE-1 bodies in Mael -/- spermatocytes by performing experiments aimed at identifying ORF1p bound RNAs in LB enriched fractions. They also perform experiments to identify novel ORF1p interactors in LBs. The authors find that LBs contain fully assembled ribosomes, yet the interaction of L1 RNA and ORF1p within LBs does not require the presence of ribosomes. They also show that ORF1p associates with PRKRA, which they demonstrate to be a novel activator of L1 retrotransposition. Overall, the manuscript is well written (with a very thoughtful discussion section, in particular) and the data is very interesting. I have a few concerns about controls used in experiments, and I am curious about a few details regarding the LBs, as detailed below.

1) RNA-seq was performed on ORF1p immunoprecipitated RNAs in Figure 3. In 3A, the authors compare the abundance of RNAs in the ORF1p IP to the RNAs in the INPUT, the TOTAL extracts and the Beads only (BO). I am worried that an important specificity control is missing, namely an IgG immunoprecipitation control. Typically, in RNA-imunoprecipitation experiments, IgG immunoprecipitation will be performed to rule out non-specific interactions. Have the authors performed this control? It seems like it is mentioned in the methods, and in supplemental tables, but I did not see it in the actual experimental analyses. The authors do confirm co-localization of two of their top hits with the ORF1p in LBs, but I am more concerned with the conclusions made from the data analyses in A-D. If the pool of immunoprecipitated RNAs is too large due to insufficient controls, then important insights could be missed, including in later figures when the length of ORF1p associated RNAs is examined.

We appreciate Reviewer's thoughts regarding the most appropriate control for immunoprecipitation (IP) and RNA-immunoprecipitation (RIP) experiments. We have carefully evaluated Beads Only (BO) and nonspecific IgG controls for both IP and RIP experiments. We evaluated the performance of both Beads Only (BO) and IgG isotype controls in an ORF1 immunoprecipitation experiment from Mael null testis extract. Subsequently, RNA-seq was performed on the immunoprecipitated material as described in the methods section. Samples were normalized by dividing read counts per gene by library size factor using DESeq2 v1.30.1. Our analysis shows that the detected mRNAs cluster in 4 groups (see dot-plot):

1) The majority of the mRNAs detected in this experiment behave similarly in BO and IgG negative controls, clustering along the best-fit line ($y = 0.77x + 115.71$, in blue) and showing either a comparable number of reads in both samples (group 1: n=12663) or no binding at all (group 2: n=5037).
2) The graph highlights two other groups: group 3 with a background only in BO (n=675) and group 4 with a background only in IgG (n=1727). These data show that both negative controls have characteristic backgrounds and that IgG doesn't perform better than BO.
3) Of 1347 mRNAs highly enriched in ORF1p immunoprecipitates from sucrose fractions 5-8 (shown in red in Fig. 3C-D), 1235 mRNAs (~92%) fall along the best fit line (groups 1 and 2), thus showing a comparable behavior in both BO and IgG negative controls. Another 29 of 1347 mRNAs fall into group 3 of RNAs binding nonspecifically to beads but not to IgGs and are accounted for in our analysis in the manuscript. Only 83 mRNA of 1347 species show some unspecific binding to IgG-coupled beads. However, the vast majority (71 out of 83) show at least a 2-fold enrichment in the corresponding generated anti-ORF1p IP sample, confirming their preferential binding to ORF1p-containing RNPs.

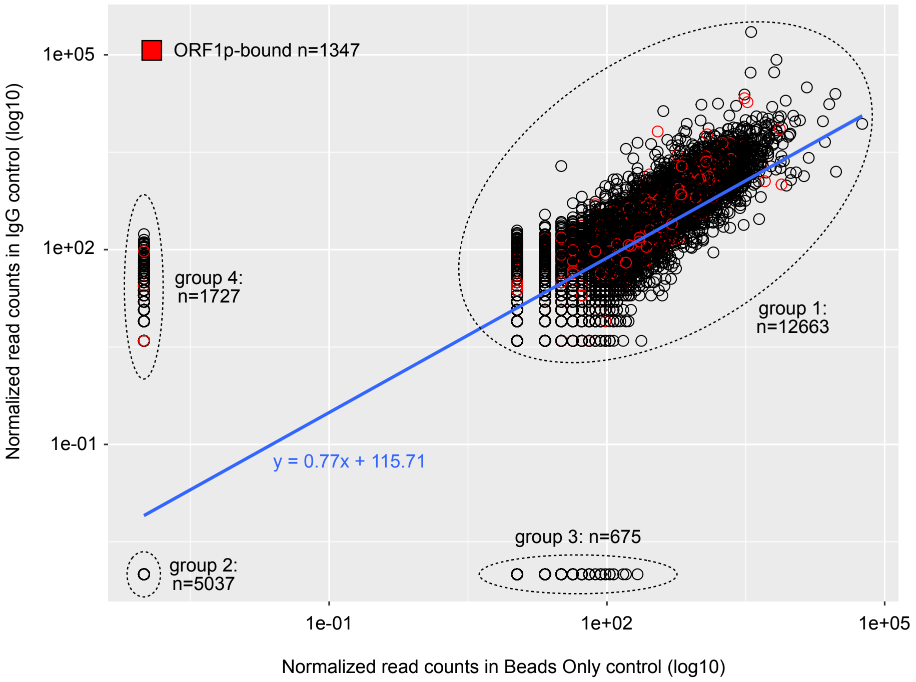

Overall, this experiment confirms the reliability of the BO negative control. We also would like to point out that, to increase the reliability and robustness of our results, we repeated the experiments with three independent biological replicates. Finally, as an additional validation, we confirmed the localization of abundant repeat and genic mRNAs within LB structures, further supporting the association between mRNPs and ORF1p. This corroboration strengthens our argument for the validity of the observed interactions.

Given these considerations, we believe the BO control was a well-justified choice for our experiments and provided a reliable and accurate assessment of nonspecific interactions in our IP/RIP studies. We are confident that our approach, coupled with the additional validations and multiple biological replicates, offers a robust and reproducible method for determining the specific interactions of interest.

2) Also, in Fig. 3A, could the authors explain why L1 RNA is more abundant in the ORF1p IP than in the INPUT? Are the input samples actually a percent of the input used in the IP experiments, or are they the remaining supernatant following IP from those fractions?

Figure 3A reports log2 normalized collapsed read counts for selected classes of genomic repeats. The data show that ORF1p immunoprecipitation enriches L1Md_T, L1 Md_A, and MMERVK10C-int RNAs compared to the starting testis lysate (TOTAL) and pooled sucrose fractions 5-8 (INPUT). In other words, there is a higher representation of L1 RNA in the pool of precipitated RNAs compared to the total pool of cellular RNAs detected in TOTAL and INPUT samples. In contrast, Beads Only (BO) control shows no enrichment.

3) On page 11, the authors make the statement that “L1 overexpression and LBs formation disrupt PBs formation.” Do the authors have any biochemical or quantitative data to support this strong statement? If not, they may want to consider rewording.

The quoted statement reflects that *Mael*$^{-/-}$ spermatocytes (but not spermatogonia) lack PBs (as identified by Ddx6 and Dcp1a staining) that are readily seen in both wild-type spermatogonia and spermatocytes. Instead, the data show the appearance of Ddx6 and, to a lesser extent, of Dcp1a in LBs. These observations suggest that Processing bodies (as defined by the cytoplasmic localization of PB markers such as Ddx6 and Dcp1a) are absent in *Mael* null spermatocytes. The cause of PB absence is unclear, but the data indicate that PBs are present initially in spermatogonia and early spermatocytes. Interestingly, in the latter, Dcp1a and ORF1p foci can be observed nearby each other, suggesting that PBs or some of their constituents might fuse with LBs.

To avoid the statement implicating ORF1p expression and LB formation with the lack of PBs, we reworded it as "...L1 overexpression and LBs formation coincided with the disappearance of PBs".

4) On page 12, the authors claim their findings "suggest that the CCR4-NOT complex does not deadenylate LB-localized mRNAs, based on their data that two ORF1p associated mRNAs, Sycp1 and Setx, exhibit no changes in polyA tail length in Mael -/- testes as compared to controls. To support their claim, the authors would need to show that CCR4-NOT associates with these RNAs in both Mael -/- and control testes.

The quoted statement reflects that despite the colocalization of CCR4-NOT complex proteins with ORF1p and mRNAs, transcriptome-wide and gene-specific analyses did not reveal evidence of RNA destabilization. It also builds on claims in the literature that SG and PB-localized mRNAs are destabilized with the participation of the CCR4-NOT complex. The question of the specific role of the CCR4-NOT complex in LBs is of great interest and will require additional analyses beyond this study's scope.

5) The EM experiments in Fig 1 are beautiful and it is interesting that the LBs from Mael -/- spermatocytes are often double membraned structures. Do the LBs in WT spermatocytes also have double membranes? Have the authors investigated whether these are some sort of ER-derived organelles? These could be interesting points to touch on in the discussion.

We agree with the Reviewer that the presence of double membranes is an intriguing feature associated with LBs. However, while the double membranes prominently associate with large LBs, our data show this is a late event during LB formation. Early small and medium-sized LBs lack such membranes, including in the wild-type germ cells. Furthermore, although the membranes are associated with large LBs, we have never observed any examples of fully membrane-enclosed LBs.

We agree with the Reviewer's suggestion that those membranes might be of ER origin. Indeed, we stained *Mael*$^{-/-}$ testis sections with antibodies to ER-resident chaperones Calreticulin and BiP. Still, we could not unequivocally assign the ER identity specifically to such double membrane structures since the signal was detected throughout the cytoplasm. Interestingly, in our Mass Spec datasets, we also noticed that ORF1p co-precipitates with BiP, further suggesting an association of LBs with the endoplasmic reticulum. Further experiments will be needed to address the partnership of LBs and double membranes.

Reviewer #2: Long Interspersed Element 1 (L1) is an active retrotransposon comprising ~18% of the mouse genome. As L1s are selfish genetic elements, they must retrotranspose in cells of the germ line or early embryonic cells, where new L1 insertions have the potential to be

inherited by subsequent generations. To curtail deleterious L1 activity, a piRNA based adaptive defence mechanism has evolved in the mouse male germ line. Numerous factors are required for piRNA biogenesis, including the RNA-binding protein Maelstrom (Mael). Mael-/- germ cells overexpress L1 ORF1p, which localizes to large cytoplasmic aggregates here termed L1 bodies, or LBs.

In this study, De Luca et al. investigated the proteins and RNAs that interact with L1 ORF1p in LBs. They find that LBs are enriched in L1 RNA, ORF1p, and ribosomes, that L1 RNA and ORF1p interact in both ribosome-associated and ribosome-independent complexes. Analysis of RNAs associated with ORF1p revealed active L1 subfamily RNAs and a variety of cellular mRNAs. IP-mass spec analysis of ORF1p interacting proteins from Mael-/- testicular extracts uncovered both known and novel interactors, including members of the CCR4-NOT complex, and strengthened the conclusion that LBs share some characteristics with but are distinct from both stress granules and P-bodies. Notably, aggregation in LBs did not appear to impact mRNA stability, poly-A tail length, or translation. Intriguingly, the RNA-independent ORF1p interactor PRKRA, which can both enhance and inhibit translation, was found to increase ORF1p levels in an ectopic overexpression system in HeLa cells.

This work is important as it characterises the L1 ORF1p protein and RNA interactome in a cell type that is relevant to the generation of heritable L1 insertions and the impact of L1 activity on genome evolution, using Mael deficiency as a model of L1 overexpression. The experiments presented are of high quality and in many ways confirm the results of previous studies, while revealing added complexity in the interaction between L1 and host factors within cytoplasmic aggregates. The most interesting implication, based on PRKAR's ability to increase L1 ORF1p levels and the finding that LBs are not associated with mRNA degradation, is that association of L1 RNPs with cellular factors in cytoplasmic inclusions, at least in germ cells, may, in part, represent an adaptive mechanism to evade host cell defences, rather than simply a sequestration of the L1 machinery by the host cell to limit retrotransposition.

Points for consideration:

1. This work entails an extensive characterization of LBs, but these structures are specific to germ cells where L1 is overexpressed due to, in this case, Mael deficiency. It would be quite interesting to examine the L1 ORF1p interactome in wild-type germ cells, which do contain ORF1p aggregates (as shown in Figure S1), in comparison to Mael-/- cells. However these experiments would be incredibly challenging given the low levels of L1 ORF1p expression in wild-type germ cells. Perhaps the authors could speculate on extent to which the molecular interactions occurring in LBs in the presence of extreme L1 overexpression might also take place in ORF1p aggregates under normal L1 expression levels. In other words, how relevant are these results to L1 activity in wild-type testis?

We thank the Reviewer for their thoughts about the potential roles of LBs in wild-type cells and the recognition of the challenges of procuring sufficient amounts of ORF1p complexes from wild-type testes. We believe we have addressed the point raised by the reviewer in the Discussion on two occasions: first, by noting the structural similarities between LBs in the mutant and wild-type germ cells and, second, by proposing that the ISR avoidance mechanism could be of significance in somatic normal and cancer cells naturally devoid of piRNAs.

2. In figure 3A, the inclusion active L1 GF subfamily elements and B1 SINEs in the heat map of repeat RNAs found in germ cell ORF1p complexes would be interesting, as these elements are also known to be active in mice.

L1Md_Gf elements are classified as L1Md_F and L1Md_T in the Repeat Masker (Sookdeo et al., 2013). L1Md_T is included in Fig. 3A, while L1Md_F showed a lower coverage than the ancestral Lx family and therefore excluded from Fig. S3A. SINE B1_Mm family has been added to Fig. 3A.

3. Can the authors speculate on the strong enrichment for MMERVK10C-int RNA in ORF1p complexes? This result is unexpected and quite interesting, yet it is not clear why this RNA would associate so strongly with ORF1p. For comparison, it would be helpful to include another active LTR retrotransposon (an IAP family for example) from the full heatmap in Figure S3, to highlight the unique enrichment for MMERVK10C-int.

Just like the Reviewer, we were surprised to see the enrichment of MMERKV10C-int RNA in ORF1p immunoprecipitates. At this point, we have no mechanistic explanation for the observation that is worthy of further examination.

Following the Reviewer's suggestion, we included RNA level data for IAPEz-int and IAPEY4_I-int families in Fig. 3A.

4. Line 71: LTR retrotransposons, not L1s, account for 10-15% of novel germline mutations in mice.

We thank the Reviewer for pointing out this error. The statement has been removed.

5. Line 344: the primary reference for the synthetic mouse L1 constructs is Han and Boeke 2004 (reference 109).

We thank the Reviewer for the suggestion. We incorporated the original reference for the synthetic mouse L1 constructs and recent study's reference that detailed the causes of L1 overexpression.

6. Line 358: a new study characterising the L1 interactome could be added to the listed references for proteomic analysis of L1 RNP interactors (PMID: 36639706).

We thank the Reviewer for the suggestion. The reference was added.

Reviewer #3: L1 ribonucleoprotein (RNP) complex is an obligatory intermediate for its replication and propagation. Many important insights about its subcellular localization and molecular composition have been gleaned from overexpressing L1 in cultured cells and most recently from prostate cancer cells by using RNA immunoprecipitation (ref 63). In this context, profiling the complexity of RNAs and their fate in the so called “L1 bodies” in mouse germ cells represents a critical effort in our understanding of not only host mechanisms of L1 regulation but also roles of L1 RNP in host gene expression. In this manuscript, De Luca et al utilized an assortment of molecular, cellular and ultracellular techniques to dissect the composition of L1 bodies and provided an initial assessment of the potential function of such complex. Overall, the experiments were well executed, and data were clearly presented, only with a few blemishes concerning some aspects of overinterpretation of the role of L1 bodies and PRKRA experiments.

Major comments:
1. The authors did an outstanding job in demonstrating L1 body morphology in Mael-/- spermatocytes with EM, the presence of selected ribosomal proteins with immunofluorescence, differential association of L1 ORF1p and polyribosome with sedimentation, the identification of TE RNA and mRNA species with RNA immunoprecipitation and sequencing, the identification of (new) protein partners interacting with L1 RNP with mass spectrometry, and the characterization of RNA integrity and translation efficiency. These experiments were carefully designed, executed, and analyzed. The comprehensive identification of RNA and protein components in L1 bodies in mouse spermatocytes is novel and lays foundation for future studies.

We thank the Reviewer for the positive evaluation of this study.

2. However, this reviewer feels that the role of L1 bodies may have been overinterpreted. As stated in the title and at the end of the abstract, the authors conclude that L1 body sustains RNA integrity and translation of endogenous RNAs in mouse spermatocytes. However, the data about the abundance of endogenous RNAs in L1 body and these RNAs' stability are correlational. Indeed, the authors also noted the caveat that these RNAs might only represent a fraction of the total amount for each RNA species. Importantly, the Mael-/- cells cannot progress through meiosis, and are not "normal" environment for L1 propagation.

We thank the Reviewer for their thoughts on the role of LBs in RNA stability and translation.

1. In response to the Reviewer's comment about a possible overinterpretation of the data, we changed the paper's title.

2. As the Reviewer correctly notes, we acknowledge the possibility of ORF1-bound mRNAs being a fraction of the total RNA content. We have attempted to address this question by determining the localization of three mRNAs enriched in ORF1p IPs. Such analysis showed an aggregation of these RNA species to LBs. Had the LB localization been detrimental to the integrity and translation of such mRNAs, we would have expected to detect at least some impairments in experiments examining poly(A) tail lengths, 5` and 3` ends abundance in RNA-seq experiments, and ribosome footprint. Therefore, the weight of the available evidence favors our proposal that the integrity and translation of LB-localized mRNAs are intact. This conclusion is further supported by our findings of avoidance of the integrated stress response activation via ORF1p-PRKRA interactions. However, we do agree with the Reviewer that further understanding of LBs and mechanisms of RNA sequestration, protection, and translation is necessary.

3. *Mael* mutant spermatocytes fail to complete spermatogenesis due to meiotic failure following pachytene checkpoint activation. However, LBs begin to develop at the onset of the meiotic prophase and are indistinguishable from those present in the wild-type testes. Therefore, the data suggest that large LBs are likely enlarged equivalents of small LBs observed in wild-type mice and could be primarily involved with the translation and assembly of L1 RNPs.

3. The authors further cauterized potential role of PRKRA by overexpressing it in HeLa cells in the presence of an L1spa reporter plasmid. They showed a slight decrease in L1spa RNA but an increase in ORF1p and L1spa retrotransposition. The authors did not check L1 body

formation and colocalization of PRKRA with ORF1p in these cells. So it remains premature to conclude that ORF1p condensate promotes L1 retrotransposition.

We thank the Reviewer for the suggestion. The relationship of LBs with L1/SG-like foci in cultured somatic cells is an important one that deserves, in our opinion, a separate study. However, in response to the Reviewer's request, we now include panel E in Fig 7, which shows the colocalization of ORF1p with PRKRA in HeLa cells.

4. In the conclusion section (page 17,18), the authors further suggest that L1 body represents an active mechanism to counteract cellular defenses as sites of active translation. This hypothesis is very interesting and quite profound but requires additional experimental support. It should be noted that these L1 bodies form in Mael-/- germ cells, which are an evolutionary dead end as they lose the capacity of differentiating into mature germ cells. So the system, while presenting abundant L1 bodies, has its limitations.

We thank the Reviewer for their thoughts on the significance of our observations. Our hypothesis represents an attempt to consider our data as a whole and to find the meaning of these observations. Our data raise the possibility that TE-host interactions are even more intricate than anticipated previously. We have been careful in our statements related to the potential implications of this study by noting their speculative nature and the need for additional future studies.

Although we relied on the piRNA mutant to reveal this phenomenon in the first place, we have successfully recreated essential aspects of our observations made in germ cells in somatic cells. Furthermore, identifying LBs in wild-type germ cells suggests that the adaptive mechanism operates under physiological conditions (albeit on a smaller scale) and could be a critical aspect of the L1 lifecycle. Dysregulation of TE defenses can also occur in animal and human populations due to the emergence of hypomorphic alleles or in response to environmental stresses, all of which could lead to increased L1 expression and retrotransposition.

Minor comments:

1. Figure 1. Some cellular landmarks should be labelled in the EM images to increase readability.

We marked nuclei (Nuc) and cytoplasms (Cyto) in EM images.

2. Figure 3A. Color key says 14, 15, and 16.

In addition to the information in the figure legend, we now show that the color key values are log2-normalized read counts corresponding to different colors in the heatmap.

3. Figure 3E. Spermatocytes and spermatids should be marked.

Spermatocytes (SPC) and round spermatids (RS) have been marked in Fig. 3E.

4. Ref 27 is misplaced.

We corrected the reference.
